# Evaluation of Potential Herb-Drug Interactions Between Shengmai Injection and Losartan Potassium in Rat and *In Vitro*

**DOI:** 10.3389/fphar.2022.878526

**Published:** 2022-04-20

**Authors:** Zhenchao Niu, Tingting Qiang, Wenyong Lin, Yiping Li, Keyan Wang, Dan Wang, Xiaolong Wang

**Affiliations:** ^1^ Branch of National Clinical Research Center for Chinese Medicine Cardiology, Shuguang Hospital Affiliated to Shanghai University of Traditional Chinese Medicine, Shanghai, China; ^2^ Cardiovascular Research Institute of Traditional Chinese Medicine, Shuguang Hospital Affiliated to Shanghai University of Traditional Chinese Medicine, Shanghai, China

**Keywords:** shengmai injections, hypertension, losartan potassium, CYP 450, drug transporters

## Abstract

**Aim:** The present study aimed to explore the potential herb-drug interactions (HDI) between Shengmai injection (SMI) and losartan potassium (LOS) based on the expression profiles of cytochromes P450 (CYP450) and drug transporters in rat and *in vitro*.

**Methods:** Different concentrations of SMI were used to explore the influence of SMI on the antihypertensive efficacy of LOS in the hypertension rat model established by N (omega)-nitro-L-arginine methyl ester (L-NAME) for 4 weeks. Subsequently, the serum concentration levels of LOS and losartan carboxylic acid (EXP3174) were determined by Liquid Chromatography Mass Spectrometry (LC-MS) and pharmacokinetic analysis. Human liver microsomes, human multidrug resistance protein 1 (MDR1/P-gp), and breast cancer resistance protein (BCRP) vesicles, human embryonic kidney 293 cell line with stable expression of the organic anion transporting polypeptide 1B1 (HEK293-OATP1B1 cells) and mock-transfected HEK293 (HEK293-MOCK) cells were used to verify the effects of SMI on CYP450 enzymes and drug transporters *in vitro*.

**Results:** Low, medium, and high concentrations of SMI increased the antihypertensive efficacy of LOS to varying degrees. The high dose SMI increased the half-life (*t*_*1/2*_)*,* the maximum plasma concentration (*C*_max_), the area under the plasma concentration-time curve (*AUC*) from time zero to the time of the last measurable plasma concentration (*AUC*_*0-t*_), *AUC* from time zero to infinity (*AUC*_*0-∞*_), and mean residence time (*MRT*) values of LOS and decreased its apparent volume of distribution (*Vd*) and clearance (*CL*) values. The *AUC*_*0-t*_ *, AUC*_*0-∞*_, and *MRT* of LOS were increased, whereas the *CL* was decreased by the medium concentration of SMI. In addition, the high, medium, and low doses of SMI increased the relative bioavailability (*Frel*) of LOS. SMI exhibited no significant effects on the pharmacokinetics of EXP3174. *In vitro*, SMI exhibited different suppressive effects on the enzyme activity levels of CYP1A2 (6.12%), CYP2B6 (2.72%), CYP2C9 (14.31%), CYP2C19 (12.96%), CYP2D6 (12.26%), CYP3A4 (3.72%), CYP2C8 (10.00–30.00%), MDR1 (0.75%), OATP1B1(2.03%), and BCRP (0.15%).

**Conclusion:** In conclusion, SMI improved the antihypertensive efficacy of LOS in the L-NAME-induced hypertension rat model by increasing the concentration of LOS, while leaving the concentration of EXP3174 intact. SMI affected the pharmacokinetic properties of LOS by decreasing the elimination of LOS. These effects might partly be attributed to the inhibition of the activities of CYP3A4, CYP2C9, and of the drug transporters (P-gp, BCRP, and OATP1B1) by SMI, which need further scrutiny.

## Introduction

Hypertension (HTN) is a major controllable risk factor for premature death and disability, which is closely associated with an increased risk of myocardial infarction, heart failure, ischemic or hemorrhagic stroke, and end-stage renal disease (Kearney et al., 2005; Mills et al., 2016). In China, the incidence of HTN is annually increasing, with one in four adults suffering from hypertension. However, its control rate is very low compared with that of the developed countries, which causes an enormous burden to the Chinese medical health system (Wang et al., 2018).

Currently, herbs are used as a complementary therapy with antihypertensive drugs. This strategy is increasingly popular in the treatment of hypertension (Zhang et al., 2020a; Van den Eynde et al., 2018). Therefore, it is vital to explore the interaction between antihypertensive drugs and herbs, since herb-drug interactions (HDI) may occur. These can be manifested as a reduced or an enhanced antihypertensive effect, or even in some cases as an increased toxic side effect (Park et al., 2019; Bin et al., 2021). Cytochrome P450 enzymes (CYPs) are highly specific phase I enzymes, which are responsible for the metabolism of 80% of the current clinical drugs and play a key role in HDI (Zanger and Schwab, 2013; De Kesel et al., 2016). Drug transporters are a class of transporters located on cell membranes that allow endogenous substances and exogenous drugs to enter and exit the cells (Estudante et al., 2013; J. P. K., 2012). Drug transporters are equally significant for HDI.

Shengmai San, a prescription of traditional Chinese medicine (TCM) originated in the Jin dynasty and is composed of Ginseng, Radix Ophiopogonis, and Schisandra (Lu et al., 2021). Shengmai injection (SMI) is an injection prepared on the basis of Shengmai San with modern drug extraction methods, which greatly improves the absorption speed and bioavailability of active ingredients. SMI has been widely used in the treatment of hypertension and chronic heart failure (Wang et al., 2020; Cao et al., 2022; Zhang et al., 2020).

As the first non-peptide angiotensin II receptor antagonist (ARB) applied clinically, losartan is widely used in the treatment of hypertension and heart failure (Pitt et al., 2000; Dahlof et al., 2002). Fourteen percentage of the total concentration of losartan is metabolized by hepatic CYP3A4 and CYP2C9 enzymes into losartan carboxylic acid (EXP3174), which is 10–40 times more potent than losartan with regard to its antihypertensive efficacy (Sica et al., 2005; Burnier and Wuerzner, 2011). In China, the combination of losartan and SMI is extremely common in patients with heart failure and hypertension, whereas certain clinical studies have shown that SMI has outstanding antihypertensive efficacy (Zhang Y. et al., 2020b; Bai et al., 2018). However, there are few studies on whether HDI occurs between losartan and SMI.

Therefore, the present study explored the influence of SMI on losartan potassium (LOS) based on three aspects including antihypertensive efficacy, pharmacokinetic characteristics, and the CYP enzyme and drug transporter activity levels. The effects of different doses of SMI on the antihypertensive efficacy and pharmacokinetic parameters of LOS and EXP3174 were explored in hypertensive rats treated with N (omega)-nitro-L-arginine methyl ester (L-NAME), a nitric oxide synthase inhibitor (van den Meiracker et al., 2002). Subsequently, the effects of SMI on the activity levels of CYP enzymes and drug transporters were further explored *in vitro*.

## Materials and Method

### Materials

SMI was purchased from Hutchison Pharmaceuticals Co., Ltd. (Shanghai, China). LOS was purchased from Merck Sharp & Dohme Limited (Kenenworth, NJ, United States). The CYP2C9 antibody was purchased from Biorbyt (Cambridge, UK), the CYP3A4 and the P-gp antibodies were purchased from Abcam (Cambridge, UK). The reference substance LOS and quinidine was purchased from Tokyo Chemical Industry Co., Ltd(Tokyo, Japan); EXP3174 was purchased from Toronto Research Chemicals Inc. (Toronto, Canada); L-NAME and irbesartan was purchased from Yuanye Biological Co., Ltd. (Shanghai, China); phenacetin, amodiaquine, diclofenac sodium, α-naphtholone, quercetin, ticlopidine, 4-hydroxydiclofenac, N-desethyl amodiaquine, dextrorphan, fluorescence Yellow, N-methyl quinidine, novobiocin, 17β-estradiol-glucoronide, rifampicin, G418 hydrochloride, buspirone, and tolbutamide were purchased from Sigma-Aldrich (St Louis, MO, United States); bupropion, ketoconazole, and acetaminophen were purchased from Tokyo Chemical Industry Co., Ltd.; cytepa and 4-hydroxymephenytoin were purchased from Sohon Chemtech Co., Ltd. (Changzhou, JS, China); hydroxybupropion was purchased from *Acanthus* Research Inc. (Mississauga, Canada); 6β-hydroxytestosterone was purchased from Zhenzhun Biotechnology Co., Ltd. (Shanghai, China). β-nicotinamide adenine dinucleotide phosphate (β-NADPH) was purchased from Roche (Basle, Switzerland); S-mephenytoin was purchased from Xianghui Pharmaceutical Technology Co., Ltd. (Shanghai, China); testosterone was purchased from Dr. Ehrenstorfer GmbH (Augsburg, Germany); dextromethorphan was purchased from Damas-beta Co., Ltd. (Shanghai, China); sulfapyrazole was purchased from Bai Lingwei Technology Co., Ltd. (Beijing, China); the transporter vesicle kit was purchased from GenoMembrane Co., Ltd. (Kanagawa, Japan).

### Preparation and Quality Control Standard of SMI

The quality control standard of SMI conformed to the National Drug Standard of the Ministry of Health of China, which specifies that the total amount of ginsenoside Rg1 and ginsenoside Re should not be lower than 0.08 and 0.04 mg in a 1 ml injection respectively, as analyzed by high-performance liquid chromatography (HPLC). the fingerprint analysis of SMI and showed that more than 10 components were determined, including ginsenoside Rg1, ginsenoside Re, ginsenoside Rf, ginsenoside Rb1, ginsenoside Rc, ginsenoside Rh1, ginsenoside Rd, schisandrin, ginsenoside Rg5 (Rk1), and ginsenoside Rh3 (Supplementary Figures S2, S3 and Supplementary Tables S8, S9).

### Animal, Human Liver Microsomes, Vesicles, and Cells

Eight-week-old 60 of SPF male Wistar rats, weighing 180–200 g, were purchased from Vital River Laboratory Animal Technology Co., Ltd. (Beijing, China). All animals were allowed free access to standard rodent feed and water in a 12:12 h light-dark cycle at an ambient temperature of 23–25°C and in a relative humidity of 40–60%. Human liver microsomes (mass concentration of 20 mg/ml) were purchased from *In Vitro* Technologies (Noble Park North, VIC, Australia); human multidrug resistance protein 1 (MDR1/P-gp) vesicles (5 mg/ml of transporter vesicle-specific protein) and breast cancer resistance protein (BCRP) vesicles (5 mg/ml of transporter vesicle-specific protein) were purchased from GenoMembrane Co., Ltd. (Kanagawa, Japan). Human embryonic kidney 293 cell line with stable expression of the organic anion transporting polypeptide 1B1 (HEK293-OATP1B1 cells) (HEK293-OATP1B1) and mock-transfected HEK293 (HEK293-MOCK) were all purchased from GenoMembrane Co., Ltd. (Kanagawa, Japan).

### Establishment of the Hypertension Rat Model and Blood Pressure Measurement

A total of 48 Wistar rats were treated with L-NAME (60 mg/kg·d^−1^) intragastrically for 4 weeks (van der Linde et al., 2003; van den Meiracker et al., 2002; Ahad et al., 2020a). Systolic blood pressure (SBP) ≥140 mmHg was considered to be a successful hypertension model and was used to maintain L-NAME treatment until animal death. The blood pressure measurement method was performed as described in previous literature studies and the blood pressure of each rat was determined by the average of 5 measurements by a non-invasive rat tail sphygmomanometer (BP-2006A, Beijing Softron Biotechnology Co., Ltd.) (Gillis et al., 2020; Dias et al., 2021). This experimental protocol was approved by the Animal Care and Use Committee of Shanghai University of Traditional Chinese Medicine.

### Determination of SMI Concentration in Rat and *In Vitro*

The clinical instructions of SMI recommended 20–60 ml/d by intravenous infusion or intravenous injection. In rat, in order to simulate the effective clinical dose as much as possible, 60, 40, and 20 ml were selected as the high, medium, and low SMI doses, respectively. Following the conversion of the dosage for the rats based on body surface area (BSA) method (Reagan Shaw et al., 2007), 6 ml/kg, 4 ml/kg, and 2 ml/kg SMI were used. A standard adult body weight of 60 kg with a total circulating blood volume of approximately 5 L results in a theoretical maximum serum concentration of 1.2% (V/V = 0.06/5*100% = 1.2%) volume fraction. According to FDA guidelines for drug interaction (DI) *in vitro* (Food and Drug Administration, 2020), the preferred highest concentration should not be lower than 10 times the maximum serum concentration at steady state, that is 12% of the volume fraction. Considering the enrichment effect in the liver, the highest concentration of the *in vitro* experiments was increased to 30.0% of the volume fraction. In order to ensure the accuracy of the IC50 fitting value, there are at least one test concentration points in every 10 times concentration (every lg concentration), and the concentration range selected for 4-6 test concentrations is generally 2–3 l g (that is 100–1,000 times). Finally, SMI concentrations of 30, 5, 2, 0.5, and 0.1% were selected.

### Effect of SMI on the Antihypertensive Efficacy of LOS in the Hypertensive Rat Model

A total of 36 hypertensive rats and 6 normal rats were included in this experiment. Hypertensive rats were randomly divided into 6 groups, including the model group without treatment, except for L-NAME; the SMI high-dose group (SMI-H group) received SMI (6 ml/kg/d) by tail vein injection; the LOS group was administered LOS (10 mg/kg/d, calculated based on BSA method) by intragastric administration; the LOS plus SMI high-dose (SMI-H + LOS), the LOS plus SMI medium-dose (SMI-M + LOS), and the LOS plus SMI low-dose groups (SMI-L + LOS) were administered SMI at 6 ml/kg/d, 4 ml/kg/d, and 2 ml/kg/d, respectively *via* tail vein injection. A total of 6 normal rats were assigned to a blank group without any treatment. Following the treatment of the rats for 2 weeks, the blood pressure of their tail artery was measured.

### Effects of SMI on the Pharmacokinetic Parameters of LOS and EXP3174 in the Hypertension Rat Model

Following the aforementioned 6 weeks of treatment, the hypertensive rats of the LOS, SMI-H + LOS, SMI-M + LOS, and SMI-L + LOS groups were sampled. Specifically, 0.5 ml blood was removed from the canthal venous plexus at 5, 15, and 30 min, and at 1, 2, 3, 4, 6, 8, 12, and 24 h following administration of the drugs. All samples were centrifuged at 3,000 rpm for 10 min, and the upper plasma was collected for Liquid Chromatography Mass Spectrometry (LC-MS) analysis.

### Effects of High-Dose SMI on the Pharmacokinetic Parameters of LOS and EXP3174 in Non-Hypertensive Wistar Rats

In order to exclude the influence of L-NAME on the pharmacokinetic profiles of LOS and EXP3174, 12 normal Wistar rats were equally divided into 2 groups. The blank plus LOS group (Blank + LOS group) received SMI (6 ml/kg/d) by tail vein injection, whereas the blank plus SMI high-dose plus LOS group (Blank + SMI-H + LOS) received SMI 6 ml/kg/d by tail vein injection combined with LOS (10 mg/kg/d) by intragastric administration. All groups were treated for 2 weeks and the blood collection time and methods of serum management were the same as those mentioned above.

### Effect of SMI on CYP Enzymes *In Vitro*

Three groups were set. In the negative control (NC), positive control (PC), and SMI groups, a blank buffer, a selective inhibitor of a specific CYP enzyme (including CYP1A2, CYP2B6, CYP2C8, CYP2C9, CYP2C19, CYP2D6, and CYP3A4), and different volume fractions of SMI (0.1, 0.5, 3.0, 10.0, 30.0%), respectively, were mixed with human liver microsomes. Following pre-incubation for 15 min, the probe substrate of each enzyme and reduced nicotinamide adenine dinucleotide phosphate (β-NADPH) was added to each group. Following co-incubation at 37°C for 30 min, the reaction was terminated by pre-cooled methanol. Following centrifugation at 1334 g for 5 min, the quantities of the metabolites produced and the relative activity of the CYP enzymes were calculated using the supernatant of the samples. The probe substrates and selective inhibitors of each CYP enzyme and the metabolites of the metabolic probe substrate are shown in Supplementary Table S1 *.*

### Effects of SMI on MDR1 and BCRP *In Vitro*

Human BCRP- and MDR1-expressing vesicles and their substrates and inhibitors, and SMI were first diluted with reaction buffer (50 mM MOPS-Tris, 70 mM KCl, and 7.5 mM MgCl2).

Refer to the method in other study (Li et al., 2022), 10 μL of vesicles were mixed with 15 μL of inhibitor or SMI, and pre-incubated at 37°C for 5 min. Then 5 μL of substrate and 20 μL of ATP were added to initiate the reaction, and incubated at 37°C for 5 min. The BCRP substrate is fluorescent yellow at a final concentration of 10 μM; the inhibitor is novobiocin at a final concentration of 100 μM. The MDR1 substrate is N-methylquinidine at a final concentration of 5 μM, and the inhibitor is ketoconazole at a final concentration of 100 μM. The final volume fraction series gradient of SMI group was 0.1, 0.5, 2, 5 and 20%. The final concentrations of BCRP and MDR1 were both 0.5 mg/ml. The final ATP concentration was 4 mM. As for BCRP and MDR1 vesicles, positive control (specific inhibitor), negative control (no inhibitor), SMI (serial gradient concentration) and passive diffusion control (no inhibitor, ATP replaced by AMP, 4 mM) were set up with 3 parallel samples per group. After the reaction system was reacted at 37°C for 5 min, 200 μL of 4°C pre-cooled stop buffer (400 mM MOPS-Tris, 700 mM KCl) was added to stop the reaction. The mixture was transferred to a 96-well filter plate and filtered with a vacuum pump. The filtrate was discarded. The Petri dishes were washed with pre-cooled buffer B2 repeatedly; Finally, 50 μL of 80% methanol was added to each well, and the filtrate was collected by centrifugation at 334 g for 2 min twice; The collected MDR1 vesicle filtrate sample was added with 100 μL of H_2_SO_4_ (0.05 mol/L), the content of N-methylquinidine was detected with a microplate reader (Ladsystems, Finland), the excitation light was 355 nm, and the emission light was 460 nm. The collected BCRP vesicle filtrate sample was added with 100 μL of DMSO solution, and the content of fluorescent yellow was detected by a microplate reader. The excitation light and emission light were 428 and 536 nm, respectively.

### Effects of SMI on OATP1B1 *In Vitro*

All cells were cultured in dulbecco’s modified eagle medium (DMEM) containing 10% fetal bovine serum, penicillin (100 U/ml), streptomycin (0.1 mg/ml) and geneticin (0.5 mg/ml), in an incubator containing 5% CO_2_ at 37°C. The cells in the logarithmic growth phase were seeded in a 24-well plate at a density of 4*10^5^ cells/well, and the transport experiment was performed when the cell confluence reached 80–90%.

The negative control (NC), positive control (PC), and SMI groups (0.1, 0.5, 2, 5, and 20%) were set up and the human-derived HEK293-MOCK cells were used as the NC group for HEK293-OATP1B1 cells. The NC group was incubated with pre-warmed blank buffer containing 17β- estradiol-glucoronide (20 μmol/L). The PC group was incubated with pre-warmed rifampicin solution (100 μmol/L) containing 17β- estradiol-glucoronide (20 μmol/L). The SMI group was incubated with pre-warmed different volume fractions of SMI (0.1, 0.5, 2, 5, and 20%) containing 17β- estradiol-glucoronide (20 μmol/L). Following incubation of the samples for 10 min, the media were discarded and 0.3 ml distilled water was added into the Petri dish. Subsequently, the mixture was frozen and thawed 3 times with liquid nitrogen to completely lyse the cells. A total of 100 μL cell lysate and the internal standard methanol was precipitated at 1:4. The samples were centrifuged at a speed of 12,010 g for 5 min at 4°C. The level of probe substrate in the supernatant was detected by LC-MS analysis in *LC-MS Methodology and Method Validation in Rat and In Vitro* Section and the protein concentration was detected by the BCA protein quantification kit (Beyotime Institute of Biotechnology, China).

### LC-MS Methodology and Method Validation in Rat and *In Vitro*

The LOS and EXP3174 in rat serum were quantitatively determined by a validated LC-MS method. The LC-MS system contained a Q/Exactive Quadrupole/Electrostatic Field Track Well High Resolution Mass Spectrometer (Thermo Fisher Scientific, Waltham, MA, United States) and a Dionex Ultimate 3,000 High-pressure liquid chromatography (Dionex, Subsidiary, CA, United States) system equipped with an electro-spray ionization source. Chromatographic separation was performed on an Acquity UPLC®HSS T3 column (2.1 mm × 100 mm, 1.8 μm) at room temperature. The mobile phase included 0.1% formic acid aqueous solution (C) and acetonitrile (D) at a flow rate of 0.3 ml/min. The proportion of phase B in the mobile phase was adjusted from 40 to 5% at 0–2 min, 5% at 2–4 min, 40% at 4–5 min. The parameters of mass spectrometry for each analyte were shown in Supplementary Figure S1 *.*

The substrates of CYP450 and OATP1B1 in rat were quantitatively determined by a validated LC-MS method. The chromatographic analysis was performed on a LC20 liquid chromatography system (Shi madzu, Kyoto, Japan). The chromatographic columns used were the DikmaInspire C18 column (50 mm × 2.1 mm, 5 μm) and the Phenomenex Synergi™ Hydro-RP 80A column (30 mm × 2 mm, 4 μm). The mass spectrometry analysis was performed on an AB Sciex API4000 mass spectrometer/liquid mass spectrometer (Applied Biosystems, Foster City, CA, United States). The mobile phase used included 0.1% formic acid aqueous solution (A) and 0.1% formic acid in acetonitrile solution (B). The proportion of phase B in the mobile phase was adjusted from 90 to 30% at 0–0.70 min, 30–5% at 0.70–0.71 min, 5% at 0.71–1.20 min, 5–90% at 1.20–1.21 min, 90% at 1.21–1.50 min, 90–30% at 1.50–1.70 min, 30% at 1.70–2.01 min, and 30–80% at 2.01–2.30 min. The parameters of mass spectrometry for each analyte were shown in Supplementary Table S2 *.*

The inter- and intra-assay accuracsy and precision, linearity, recovery, matrix effects and stability of the quality control samples for each analyte were <15% (Supplementary Tables S3–S5). More details were shown in Supplementary Data.

### Western Blot Analysis

A total of 100 mg rat liver tissue was mixed with 1 ml radio immunoprecipitation assay (RIPA) and 10 μL phenylmethylsulfonyl fluoride (PMSF). The tissues were ground thoroughly with a grinder and centrifuged at 12,000 r/min at 4°C for 20 min. Subsequently, the supernatant was collected and the BCA protein assay kit was used to determine the concentration of the total protein. A total of 10 μg denatured total protein samples were separated and transferred to a nitrocellulose membrane using Trans-Blot Turbo system (Bio-Rad, Hercules, CA, United States). The membranes were blocked by 5% skimmed milk powder for 1 h and subsequently incubated with the primary antibody overnight at 4°C. The blots were washed and incubated with the secondary antibody at room temperature for 1 h. The immunoreactive bands were detected by chemiluminescence.

### Pharmacokinetic Analysis and IC_50_ Value Determination

The collected data were analyzed through Xcalibur 4.1 software. The serum drug concentration data in each sample were obtained by calculation. The pharmacokinetic parameters corresponding to each rat were calculated using the “non-compartmental model” of the “PK solution 2.0TM (Summit Research Services, United States)” pharmacokinetic software. The half-life (*t*_*1/2*_), peak drug concentration (*C*_max_), peak time (*T*_max_), the area under the plasma concentration-time curve (*AUC*) from time zero to the time of the last measurable plasma concentration (*AUC*_*0-t*_), *AUC* from time zero to infinity (*AUC*_*0-∞*_), mean residence time (*MRT*), clearance (*CL*), apparent volume of distribution (*V*_*d*_), and relative bioavailability (*Frel*) were calculated. The half maximal inhibitory concentration (IC_50_) values were determined by GraphPad Prism 8.

### Statistical Analysis

The statistical analysis of the data was performed on SPSS 21.0 and the measurement data conforming to normal distribution were expressed as mean ± standard deviation. The blood pressure and the pharmacokinetic data of each group were compared by one-way analysis of variance (ANOVA). The effects of the SMI, NC, and PC groups on the activity levels of the CYPs and the drug transporters were analyzed by the two-sample t-test. The analysis of variance was used to compare the effects on each CYP enzyme and drug transporter following treatment of the animals with the corresponding SMI concentration. *p* < 0.05 was considered to indicate statistically significant differences.

## Result

### SMI Increased the Antihypertensive Efficacy of LOS

**FIGURE 1:**
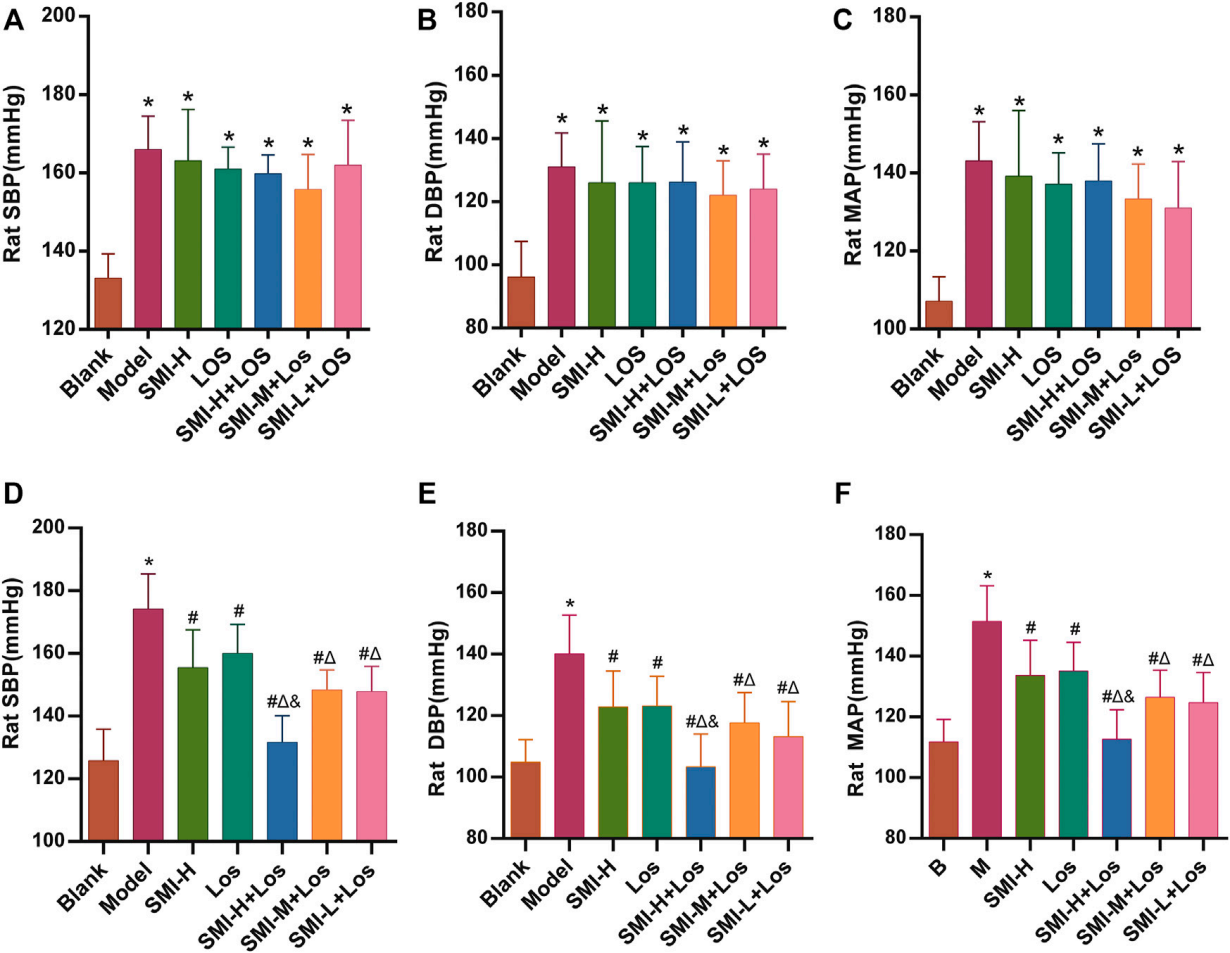
The SBP, DBP and MAP of each group were shown after 4 weeks **(A–C)** and 6 weeks **(D–F)** treatment. * indicates *p* < 0.05 compared with the Blank group. # indicates *p* < 0.05 compared with the model group; Δ indicates *p* < 0.05 compared with the LOS group; and indicates *p* < 0.05 compared with the SMI-M + LOS and SMI-L + LOS group. Model group was treated with L-NAME 60 mg/kg·d-1 intragastrically; the SMI high-dose group (SMI-H group) received SMI 6 ml/kg/d in tail vein injection; LOS group was given LOS 10 mg kg^−1^. d^−1^ intragastrically; LOS plus SMI high-dose group (SMI-H + LOS group), LOS plus SMI medium-dose group (SMI-M + LOS group) and LOS plus SMI low-dose group (SMI-L + LOS group) were respectively given SMI 6 ml kg^−1^. d^−1^, 4 ml kg^−1^. d^−1^ and 2 ml kg^−1^. d^−1^ in tail vein injection, based on LOS 10 mg kg^−1^. d^−1^ intragastrically. SBP, systolic blood pressure; DBP, diastolic blood pressure; MAP, mean arterial pressure; L-NAME, N (omega)-nitro-L-arginine methyl ester.

Following 4 weeks of L-NAME treatment, the SBP, diastolic blood pressure (DBP), and mean artery pressure (MAP) activity levels of the hypertension model group were increased by 37.83 ± 8.52, 34.83 ± 8.82, and 36.00 ± 6.10 mmHg respectively (*p* < 0.05) (Figures 1A–C) The SBP of each rat in the hypertension model group was higher than 140 mmHg. Following 6 weeks of continuous administration of L-NAME, the hypertension model group exhibited an average increase in SBP (50.50 ± 6.09) mmHg, an average increase in DBP (42.33 ± 15.34) mmHg, and an average increase in MAP (46.00 ± 13.11) mmHg.

The SBP, DBP, and MAP of each group were determined. The data indicated that in the LOS, SMI-H, SMI-H + LOS, SMI-M + LOS, and SMI-L + LOS groups these parameters were increased compared with the corresponding values noted in the model group (*p* < 0.05) (Figures 1D–F). The degree of decrease in SBP, DBP, and MAP in the SMI-H group was 10.9, 12.9, and 11.9%, respectively (*p* < 0.05). The percentages of these parameters were further decreased in the SMI-H + LOS, SMI-M + LOS, and SMI-L + LOS groups compared with those of the LOS group (*p* < 0.05). The SBP, DBP, and MAP of the SMI-H + LOS group were lower than those noted in the SMI-H, SMI-M + LOS, and SMI-L + LOS groups (*p* < 0.05). The SBP, DBP, and MAP of the SMI-H + LOS group were decreased by 24.7, 26.4, and 25.8%, respectively compared with the corresponding values noted in the model group.

### SMI Increases the Serum Concentration of LOS but Not of EXP3174

**FIGURE 2:**
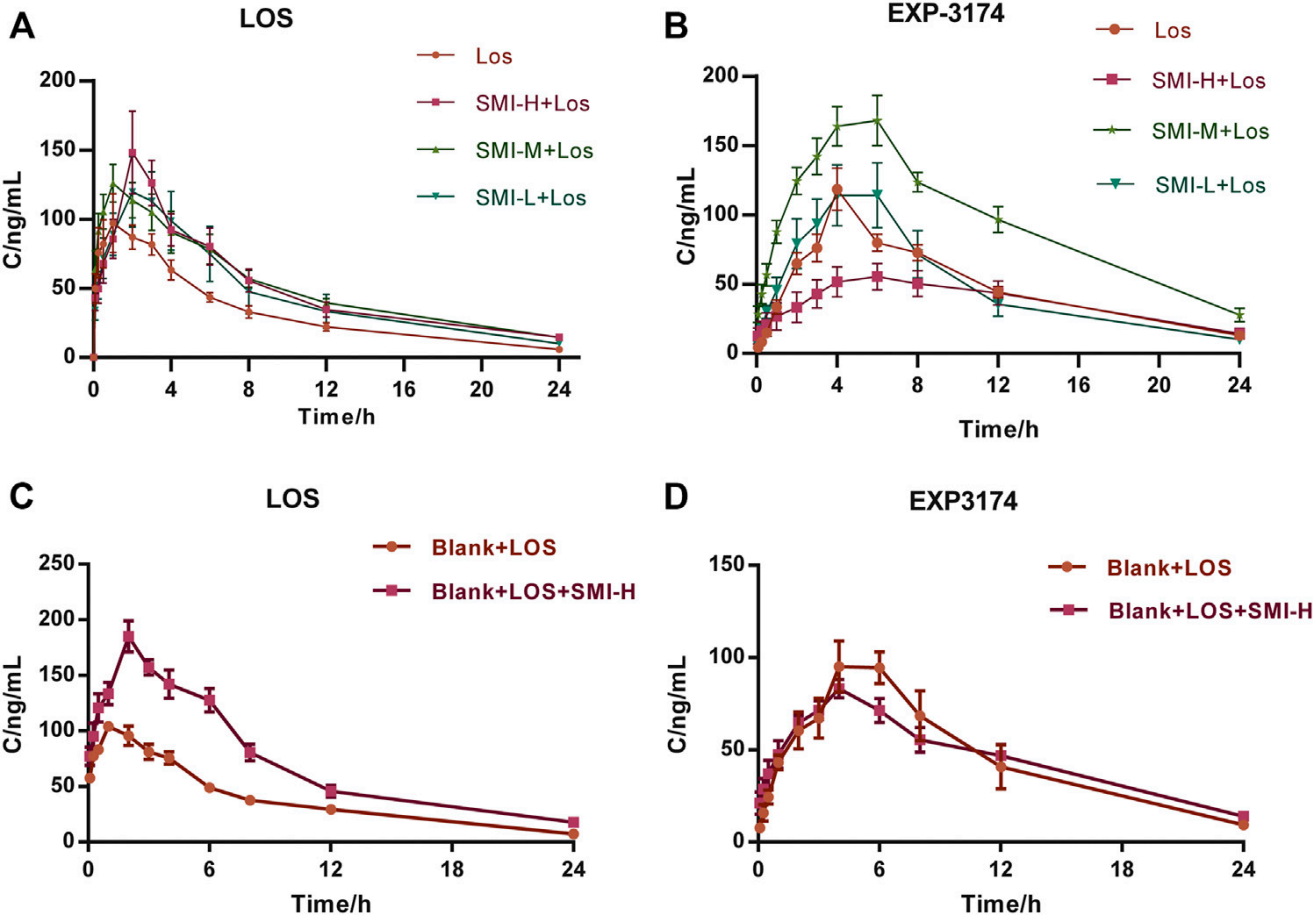
The concentration-time curves of LOS and EXP3174 with **(A,B)** and without **(C,D)** L-NAME treatment. Model group was treated with L-NAME 60 mg kg^−1^. d^−1^ intragastrically; SMI high-dose group (SMI-H group) received SMI 6 ml kg^−1^. d^−1^ in tail vein injection; LOS group was given LOS 10 mg kg^−1^. d^−1^ intragastrically; LOS plus SMI high-dose group (SMI-H + LOS group), LOS plus SMI medium-dose group (SMI-M + LOS group) and LOS plus SMI low-dose group (SMI-L + LOS group) were respectively given SMI 6 ml kg^−1^. d^−1^, 4 ml kg^−1^. d^−1^, 2 ml kg^−1^. d^−1^ in tail vein injection, based on LOS 10 mg kg^−1^. d^−1^ intragastrically. Blank + LOS received LOS 10 mg kg^−1^. d^−1^ by intragastric administration without L-NAME treatment; Blank + LOS + SMI-H group received SMI 6 ml/kg/d in tail vein injection and LOS 10 mg kg^−1^. d^−1^ by intragastrically administration without L-NAME treatment. LOS, losartan potassium; EXP3174, losartan carboxylic acid; L-NAME, N (omega)-nitro-L-arginine methyl ester.

The *t*_*1/2*_ values of the SMI-H + LOS, SMI-M + LOS, and SMI-L + LOS groups were 9.92 ± 0.96, 8.41 ± 1.43, and 6.74 ± 0.64 h, respectively, which were higher than those noted in the LOS group (6.19 ± 0.43 h) (Table 1). However, significant differences were noted only in the SMI-H + LOS group compared with the LOS group. The *C*_max_ values of the SMI-H + LOS, SMI-M + LOS, and SMI-L + LOS groups were 155.88 ± 64.09, 131.85 ± 34.99, and 125.38 ± 53.56 ng/ml, respectively, which were higher than those of the LOS group (111.78 ± 41.67 ng/ml). However, the results were not statistically significant. The *AUC*_*0-t*_ values of the SMI-H + LOS, SMI-M + LOS, and SMI-L + LOS groups were increased by 58.1, 58.2, and 40.1%, respectively compared with those of the LOS group (*p* < 0.05). In addition, the *MRT* values of the SMI-H + LOS and SMI-L + LOS groups were increased (*p* < 0.05), and the *V*_*d*_ corresponding values were decreased compared with those of the LOS group (*p* < 0.05). In the SMI-H + LOS group, the *CL* value of LOS was also lower than that of the LOS group (*p* < 0.05). No significant differences were noted in the pharmacokinetic parameters between the SMI-L + LOS and the LOS groups. In addition, the relative bioavailability (*Frel*) of LOS in the SMI-H + LOS, SMI-M + LOS, and SMI-L + LOS groups were 157.98, 158.19, and 140.85%, respectively. The concentration-time curve of LOS and EXP3174 is shown in Figures 2A,B

**TABLE 1:** The pharmacokinetic parameters of LOS in each group with L-NAME treatment. *i indicates *p* < 0.05 compared with LOS group.

Parameter	LOS Group	SMI-H+LOS Group	SMI-M+LOS Group	SMI-L+LOS Group
*t*_*1/2*_(h)	6.19 ± 0.43	9.92 ± 0.96*	8.41 ± 1.43	6.74 ± 0.64
*C*_max_ (ng/ml)	111.78 ± 41.67	155.88 ± 64.09	131.85 ± 34.99	125.38 ± 53.56
*T*_max_(h)	2 ± 0.89	2.33 ± 0.52	1.5 ± 0.55	2.2 ± 0.84
*AUC*_*0-t*_ (ng/ml.min)	787.48 ± 213.34	1,244.03 ± 379.75*	1,245.75 ± 236.12*	1,109.14 ± 673.91
*AUC*_*0-∞*_(ng/ml.min)	841.02 ± 232.49	1,417.72 ± 469.46*	1,427.72 ± 247.68*	1,210.54 ± 776.67
*MRT*(h)	8.18 ± 0.89	12.07 ± 1.02*	11.28 ± 2.22*	8.54 ± 1.80
*Vd* (mL/kg)	282,441.65 ± 73,729.85	277,463.55 ± 98,140.99	217,016.60 ± 45,076.20	264,405.76 ± 142,122.69
*CL* (ml/h/kg)	32,042.39 ± 10,551.59	19,127.96 ± 5,549.27*	17,964.40 ± 3,185.66*	27,497.47 ± 14,702.73
*Frel* (%)	——	157.98	158.19	140.85

The *C*_max_ of EXP3174 was decreased in the SMI-H + LOS group compared with that of the LOS group (*p* < 0.05). No significant differences were noted in the other indices between these two groups (*p* > 0.05) (Table 2). The drug-timing curve of LOS and EXP3174 is shown in Figures 2C,D

**TABLE 2:** The pharmacokinetic parameters of EXP3174 with 6 weeks L-NAME treatment. * indicates *p* < 0.05 compared with LOS group.

Parameter	LOS Group	SMI-H+LOS Group	SMI-M+LOS Group	SMI-L+LOS Group
*t*_*1/2*_(h)	6.76 ± 1.45	7.31 ± 1.62	6.59 ± 1.44	6.67 ± 1.34
*C*_max_ (ng/ml)	119.03 ± 36.40	81.15 ± 8.14*	123.24 ± 44.64	132.64 ± 42.82
*T*_max_ (h)	4.33 ± 0.82	6 ± 1.26	5.33 ± 1.03	4.8 ± 1.10
*AUC*_*0-t*_ (ng/ml.min)	1,160.77 ± 205.21	1,160.77 ± 205.21	1,377.60 ± 425.01	1,181.08 ± 489.10
*AUC*_*0-∞*_ (ng/ml.min)	1,296.33 ± 243.83	1,377.06 ± 218.44	1,500.78 ± 408.15	1,281.28 ± 537.94
*MRT* (h)	11.15 ± 2.34	13.28 ± 2.66	11.42 ± 1.62	9.50 ± 1.80
*Vd* (ml/kg)	175,713.60 ± 57,295.84	188,473.60 ± 36,260.16	159,469.35 ± 65,037.84	226,676.52 ± 129,635.61
*CL* (ml/h/kg)	19,842.49 ± 3,602.55	18,577.68 ± 3,249.85	17,618.36 ± 4346.02	23,408.16 ± 11,602.26
*Frel* (%)	——	101.88	118.68	101.75

**FIGURE 3:**
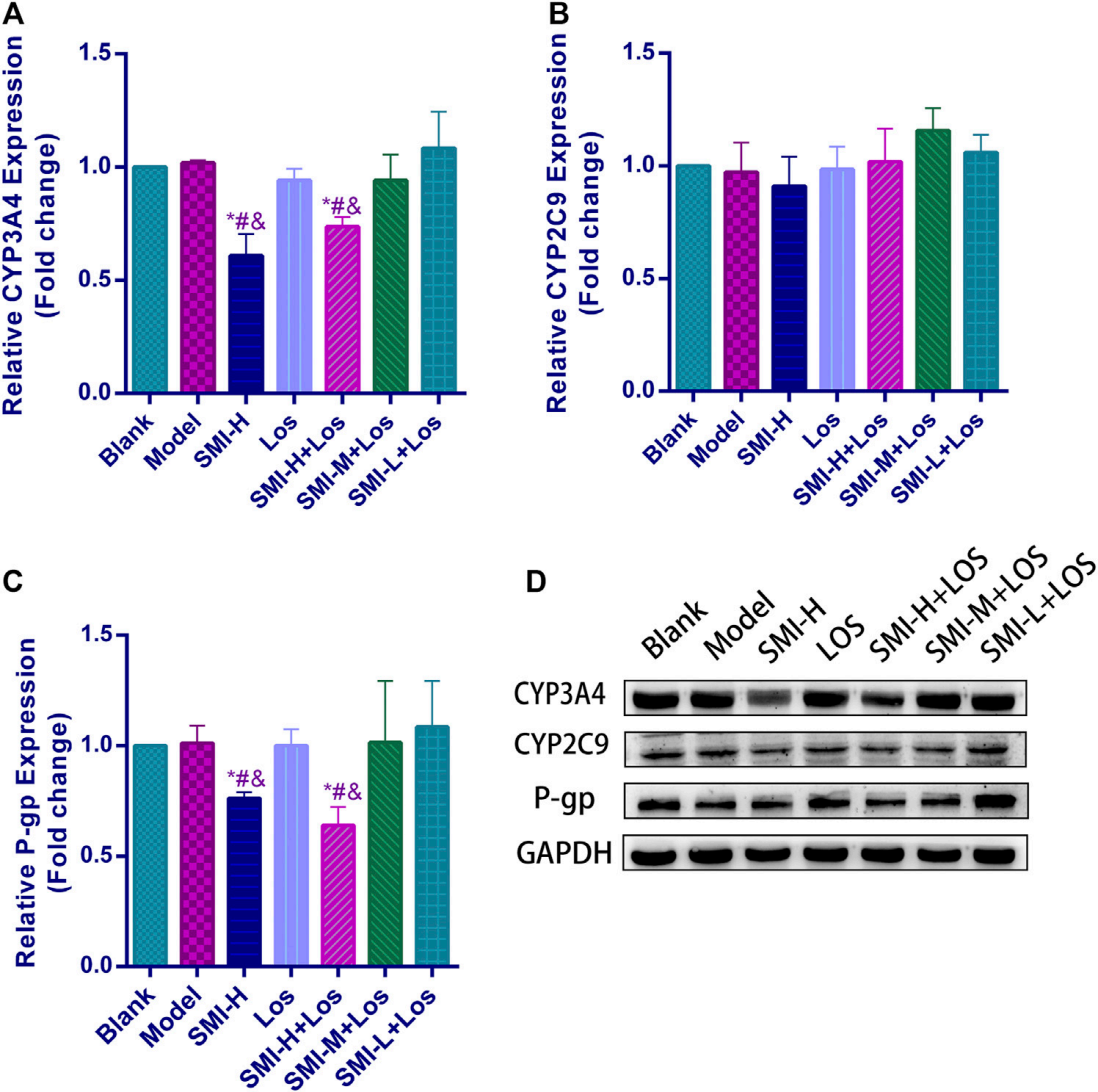
The relative protein expression levels of CYP3A4 **(A)**, CYP2C9 **(B)** and P-gp **(C)** and protein bands of these **(D)** in each group with L-NAME treatment. * indicates *p* < 0.05 compared with Blank group; # indicates *p* < 0.05 compared with Model group; and indicates *p* < 0.05 compared with LOS group. Model group was treated with L-NAME 60 mg kg^−1^. d^−1^ intragastrically; SMI high-dose group (SMI-H group) received SMI 6 ml kg^−1^. d^−1^ in tail vein injection; LOS group was given LOS 10 mg kg^−1^. d^−1^ by intragastric administration; LOS plus SMI high-dose group (SMI-H + LOS group), LOS plus SMI medium-dose group (SMI-M + LOS group) and LOS plus SMI low-dose group (SMI-L + LOS group) were respectively given SMI 6 ml kg^−1^. d^−1^, 4 ml kg^−1^. d^−1^, 2 ml kg^−1^. d^−1^ in tail vein injection, based on LOS 10 mg kg^−1^. d^−1^ intragastrically.

In normal Wistar rats, the t_1/2_, *C*_max_, *AUC*_*0-t*_, *AUC*_*0-∞*_ and *MRT* values of the BLANK + SMI-H + LOS group were increased compared with those of the BLANK + LOS group (*p* < 0.05), while those of *Vd* and *CL* were decreased (*p* < 0.05) (Table 3). The *C*_max_ values of EXP3174 were decreased in the BLANK + SMI-H + LOS group compared with those of the blank-LOS group (*p* < 0.05), while no significant differences were noted in the other indices. The concentration-time curve of LOS and EXP3174 are shown in Figure 3.

**TABLE 3:** The pharmacokinetic parameters of LOS and EXP3174 in Wistar rat without L-NAME.

Parameter	LOS		EXP3174	
	BLANK + LOS Group	BLANK + SMI-H+LOS Group	BLANK + LOS Group	BLANK + SMI-H+LOS Group
*t*_*1/2*_ (h)	6.03 ± 0.19	8.76 ± 1.08*	6.50 ± 1.52	6.84 ± 1.31
*C*_max_ (ng/ml)	107.53 ± 6.26	184.93 ± 34.05*	114.67 ± 12.13	85.2 ± 9.03*
*T*_max_ (h)	1.67 ± 0.52	2	5.33 ± 1.03	4.67 ± 1.03
*AUC*_*0-t*_ (ng/ml.min)	911.33 ± 115.08	1,699.42 ± 264.08*	1,019.3 ± 467.11	1,084.8 ± 230.95
*AUC*_*0-∞*_ (ng/ml.min)	974.77 ± 120.57	1928.07 ± 325.07*	1,170.23 ± 487.54	1,228.92 ± 273.77
*MRT* (h)	8.6 ± 0.54	10.63 ± 1.75*	9.77 ± 0.26	11.53 ± 2.29
*Vd* (ml/kg)	90,573.23 ± 13,394.06	67,493.75 ± 17,745.97*	99,589.9 ± 60,294.87	83,192.12 ± 19,569.52
*CL* (ml/h/kg)	10,392.48 ± 1,296.50	5,327.27 ± 1,010.94*	9860.44 ± 3,981.60	8,667.18 ± 2,878.72

Treatment. * indicates *p* < 0.05 compared with LOS group.

### SMI Inhibits the Activity Levels of CYP3A4 and P-Gp but Not of CYP2C9 in the Liver Tissues of Hypertensive Rats

The CYP2C9 expression levels of the blank, model, SMI-H, LOS, SMI-H + LOS, SMI-M + LOS, and SMI-L + LOS groups indicated no significant differences (*p* > 0.05) (Figure 3). The CYP3A4 expression levels in the SMI + H and SMI-H + LOS groups were lower than those noted in the blank, model, LOS, SMI-M + LOS, and SMI-L + LOS groups (*p* < 0.05). No significant differences were noted in the expression levels of P-gp between the blank and model groups (*p* > 0.05). The expression levels of P-gp in the SMI-H and SMI-H + LOS groups were decreased compared with those of the model group (*p* < 0.05).

### Inhibitory Effects of SMI on CYP Enzymes and Drug Transporters *In Vitro*

**FIGURE 4:**
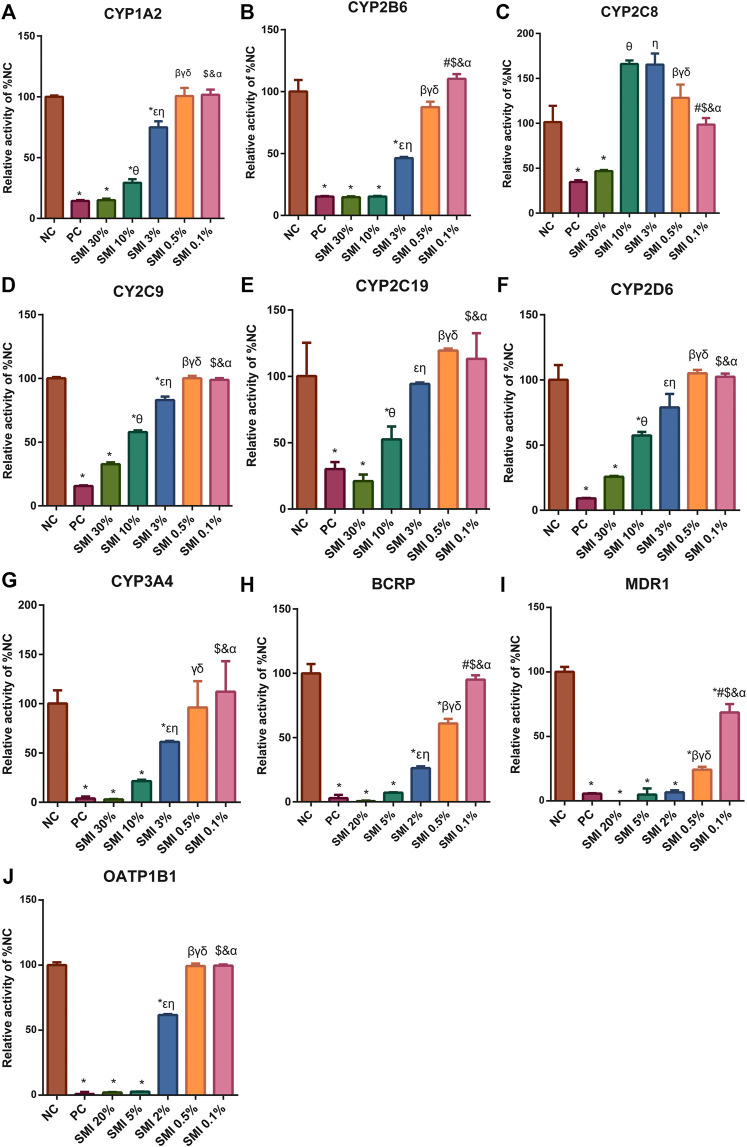
After different concentrations of SMI treatment, the CYP450 enzyme **(A–G)** and drug transporters **(H–J)** activities were shown. * indicates *p* < 0.05 compared with NC group. Comparison of enzyme activities after different concentrations of SMI treatment, in figure **(A–G)**, 0.1% *vs*. 0.5%, #*p* < 0.05; 0.1% *vs*. 3%, $*p* < 0.05; 0.1% *vs*. 10%, &*p* < 0.05; 0.1% *vs*. 30%, αp<0.05; 0.5 *vs*. 3%, βp<0.05; 0.5 *vs*. 10%, γp<0.05; 0.5 *vs*. 30%. δp<0.05; 3 *vs*. 10%, εp<0.05; 3–30%, ηp<0.05; 10–30%, θp<0.05. In figure **(H–J)**, 0.1 *vs*. 0.5%, #*p* < 0.05; 0.1 *vs*. 2%, $*p* < 0.05; 0.1 *vs*. 5%, &*p* < 0.05; 0.1 *vs*. 20%, α*P*<0.05; 0.5 *vs*. 2%, β*P*<0.05; 0.5 *vs*. 5%, γ*P*<0.05; 0.5 *vs*. 20%, δ*P*<0.05; 2 *vs*. 5%, ε*P*<0.05; 2 *vs*. 20%, η*P*<0.05; 5 *vs*. 20%, θ*P*<0.05. NC, negative control group; PC, positive control group.

**FIGURE 5:**
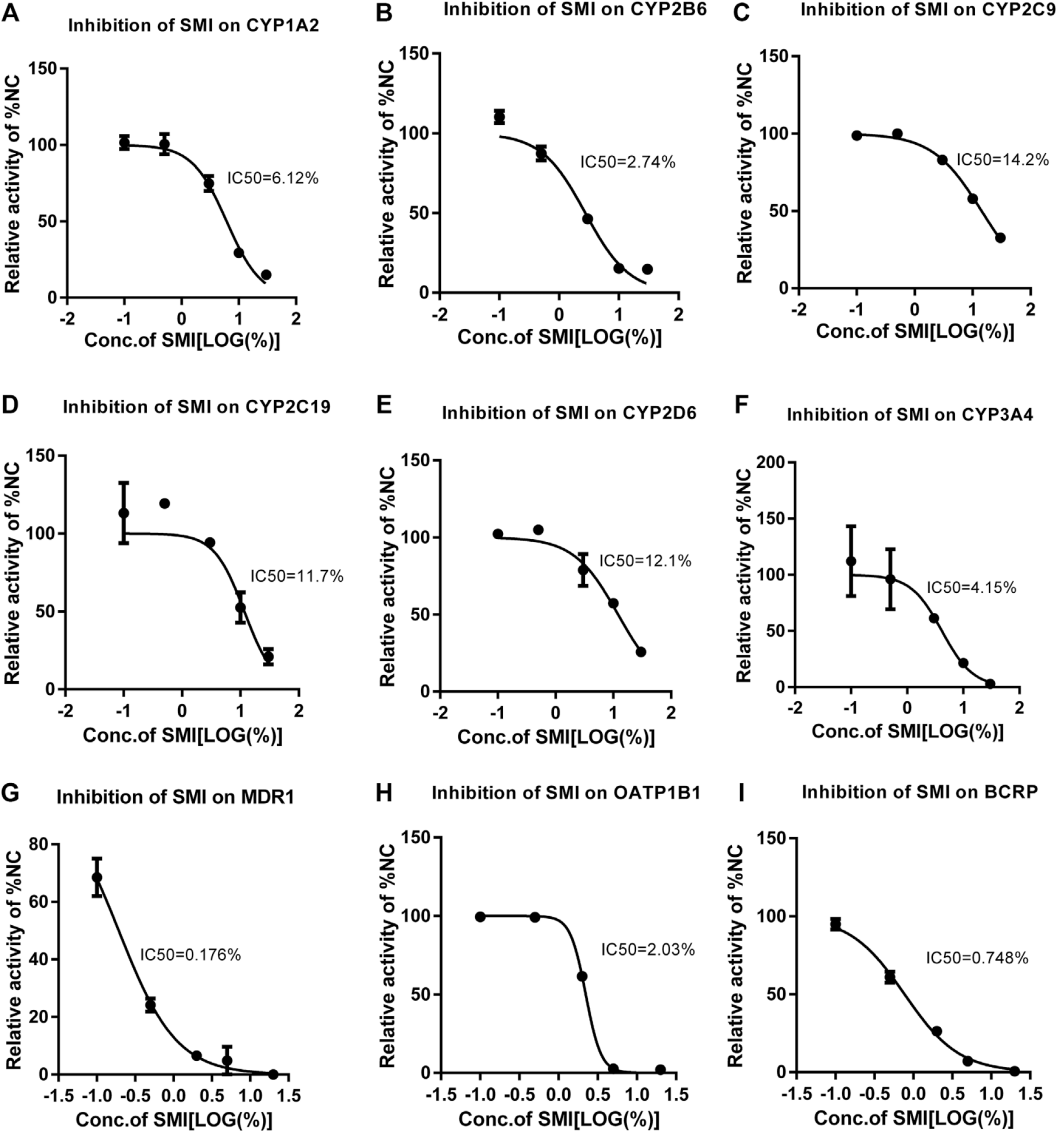
Different concentrations of SMI correspond to different CYP450 enzyme activities and the IC50 curve. X is the logarithm of SMI concentration; Y is the corresponding percentage of NC. The nonlinear formula, “Y = 100/(1 + 10^((X-logic50))" (Y starts at Bottom and goes to Top with a sigmoid shape). The results showed that SMI had different inhibitory effects on CYP1A2 **(A)**, 2B6 **(B)**, 2C9 **(C)**, 2C19 **(D)**, 2D6 **(E)**, 3A4 **(F)**, MDR1 **(G)**, BCRP **(H)**, OATP1B1**(I)**. As for CYP2C8, since SMI increased CYP2C8 metabolism in the no/weak inhibition interval, inhibition was only observed at high concentrations. When it is not clear why SMI increases CYP2C8 metabolism in the no/weak inhibition interval, the current test system cannot determine a more accurate IC50. Therefore, the data cannot fit the IC50 curve. We can only be sure that the IC50 value is between 10 and 30%.

In liver microsomes, SMI (0.1, 0.5, 3.0, 10.0, 30.0%) exhibited a dose-dependent inhibitory effect on the enzyme activities of CYP1A2, CYP2B6, CYP2C9, CYP2C19, CYP2D6, and CYP3A4 (Figures 4A–G). The IC_50_ values were estimated to be 6.12, 2.72, 14.31, 12.96, 12.26, and 3.72%, respectively (Figures 5A–F). SMI inhibited the activity of the CYP2C8 enzyme in human liver microsomes, with IC_50_ values ranging from 10.00 to 30.00%. The details of the relative activity levels of the various CYPs are shown in Supplementary Table S6.

In the MDR1 and BCRP vesicles, the uptake rates of n-methylquinidine and fluorescent yellow probe substrates in the negative control group were 14.2 and 20.4, respectively (Figures 4H,I). The transport activity levels of MDR1 and BCRP were inhibited by 98.5 and 97.1%, respectively, indicating that the model was suitable for this experiment. SMI (0.1, 0.5, 2.0, 5.0, and 20.0%) inhibited BCRP transport of fluorescent yellow and MDR1 transport of N-methylquinidine in a dose-dependent manner, with IC_50_ values of 0.75 and 0.15%, respectively (Figures 5G,H). The detailed activity levels of MDR1 and BCRP are shown in Supplementary Table S7.

In HEK293 cells, the uptake rate of the probe substrate mediated by the OATP1B1 transporter was 23.9 in the negative control group, and the transport activity of the substrate in the positive control group was reduced to 0.889% of that in the negative control group following the addition of selective inhibitors (Figure 4J). This indicated that the test system of the transporter was suitable for this experiment. SMI (0.1, 0.5, 2.0, 5.0, and 20.0%) inhibited OATP1B1 transport activity in a dose-dependent manner, with an IC_50_ value of 2.03% (Figure 5I). The detailed relative activity levels of OATP1B1 are shown in Supplementary Table S7 ***.***

## Discussion

In the present study, the effects of SMI were investigated on the antihypertensive efficacy of LOS. Moreover, the changes of the pharmacokinetic characteristics of LOS and EXP3174 and the effects of SMI on the expression levels of CYP3A4, CYP2C9, and P-gp were assessed in the liver. Furthermore, the direct inhibitory effects of SMI on certain CYP subtypes and common drug transporters in the liver were verified *in vitro*. Following 4 weeks of L-NAME treatment, the SBP, DBP, and MAP of Wistar rats indicated a significant increase, and following 6 weeks of treatment, an upward trend was evident, which was consistent with the results reported in similar studies (Chen et al., 2019). Apparently, the SMI indicated antihypertensive efficacy as expected. Notably, the SBP, DBP, and MAP of the SMI-H group were decreased by 10.92, 12.86, and 11.92%, respectively. It was the first time that the antihypertensive effect of SMI was observed in the L-NAME-constructed hypertensive rat model.

The SBP, DBP and MAP of the LOS group were decreased by 6.32, 12.14, and 11.92% compared with the corresponding values of the model group, respectively. These effects were equivalent to the antihypertensive efficacy of a high-dose SMI. However, previous similar studies demonstrated that SBP was decreased by 14.7% within 2 h following administration of 10 mg kg^−1^ LOS in the hypertension rat model established following 2 weeks of animal treatment with L-NAME (Ahad et al., 2020b). Surprisingly, following 2 weeks of combined treatment of LOS with SMI, the L-NAME-induced elevated blood pressure was further alleviated on the basis of LOS. This effect was noted for all doses of SMI used. Moreover, the SMI-H + LOS group indicated the highest hypotensive amplitude with SBP, DBP, and MAP decreasing by 24.71, 26.43, and 25.83%, respectively. Although LOS is a classic angiotensin II type 1 receptor **(**AT1R) receptor inhibitor, the mechanism of SMI lowering blood pressure is still unclear. Therefore, we cannot accurately determine whether the antihypertensive effect produced by the combination of the two is a simple additive effect or a synergistic effect based on blood pressure values.

The metabolism of LOS may be affected by other inhibitors or inducers. Ahad et al. demonstrated that in the L-NAME-induced hypertension rat model, losartan combined with *Hibiscus sabdariffa* or *Zingiber officinale* could increase the concentration of this drug in the serum and enhance its antihypertensive efficacy (Ahad et al., 2020b). Similarly, amlodipine, an antihypertensive drug that was metabolized primarily by CYP3A4, increased the serum exposure to losartan, thereby increasing its antihypertensive efficacy (Park et al., 2019). Therefore, we hypothesized that the antihypertensive effect produced by SMI combined with LOS might be a synergistic effect, resulting from the increased serum concentrations of LOS or EXP3174 following SMI use.

SMI inhibits the metabolism of LOS. It was found that the *AUC*_*0-t*_ values of LOS in the SMI-H + LOS, SMI-M + LOS, and SMI-L + LOS groups were increased by 39.45, 17.95, and 12.17%, respectively compared with those of the LOS group, indicating that SMI increased the concentration of LOS in the serum in a dose-dependent manner. According to the FDA standards, a two-fold increase noted in the average AUC value corresponds to a medium-potency inhibition, indicating that SMI had not reached this type of activity (Huang et al., 2008). The relative bioavailability of LOS was improved following its combination with SMI, indicating that the latter could promote the absorption of LOS in the gastrointestinal tract. SMI may inhibit the activities of CYP3A4, CYP2C9 and P-gp in the intestinal tract leading to a reduction in the metabolism and efflux of LOS. Choi et al. studied the interaction between licochalcon A and losartan, and demonstrated that licochalcon A could increase the relative bioavailability of LOS, which was similar to the results of the present study (Choi et al., 2013). Similarly, Ma et al. demonstrated that Xuesaitong injection could also enhance the relative bioavailability of losartan without additional interpretation (Ma et al., 2019).

SMI did not increase the serum concentration of EXP3174, whereas the *C*_max_ of the SMI-H + LOS group was lower than that of the LOS group. The *C*_max_ of EXP3174 indicated a dose-dependent trend of SMI, although no significant differences were noted between the SMI-M + LOS and the SMI-L + LOS groups. In previous studies, the effects of other drugs were assessed on losartan metabolism, whereas the serum concentration of EXP3174 exhibited diverse results, which were characterized as reduced, unchanged, or increased. Dong et al. indicated that Ginkgo leaf tablets could reduce the serum concentration of EXP3174 and increase the serum concentration of LOS. The *AUC*_*0-t*_ of LOS was increased by 70% and this increase was higher than that of SMI (Dong et al., 2018). Choi et al. demonstrated that licochalcon A increased the serum concentration of LOS without affecting the serum concentration of EXP3174. The *AUC* of LOS was increased by 33.38%, which was consistent with the results of the present study (Choi et al., 2013). Chen et al. indicated that Glimepirea could increase the concentration levels of LOS and EXP3174 in the serum. The *AUC*_*0-t*_ of LOS was increased by 11.18%, which was lower than that of SMI (Chen et al., 2015). The serum concentration of EXP3174 was associated with the degree of inhibition of CYP3A4 and CYP2C9 based on the fact that SMI could only inhibit the activity levels of the metabolic enzymes CYP3A4 and CYP2C9. The serum concentration of EXP3174 indicated a dose-dependent trend following the increase in SMI dosage, which was contradictory to the initial results of the present study.

SMI could inhibit the metabolism of LOS in Wistar rats without L-NAME treatment, whereas it exhibited no significant effects on the metabolism of EXP3174, which was consistent with the results of the hypertension rat model. It was noted that the influence of L-NAME on the CYP3A4 and CYP2C9 enzyme activities was negligible, which further confirmed the inhibitory effects of SMI on LOS metabolism.

The metabolism of LOS in the liver is the pivotal process affecting drug efficacy, in which the CYP enzymes and the drug transporters are the primary enzymes involved. Therefore, we selected specific members of drug detoxification enzyme classes to comprehensively and intuitively assess the effects of SMI on CYP enzymes and drug transporters in the liver. Specifically, the following members were examined: P-gp, BCRP, OATP1B1, CYP3A4, and CYP2C9. These enzymes, which are mainly present in hepatocytes, were selected to carry out extracorporeal experiments, which may explain the alteration in the pharmacokinetic properties of LOS and EXP3174 following the combined treatment of SMI and LOS *in vivo* (Huang et al., 2008). LOS was not selected as a probe substrate for CYP3A4 or CYP2C9 enzymes, since LOS was a non-specific probe substrate, which could be simultaneously metabolized by two CYP enzymes. This could not explain the inhibitory effect of SMI on a single CYP enzyme.

SMI exhibited inhibitory effects on CYP3A4 and CYP2C9 *in vitro*, which metabolize LOS. It also inhibited P-gp, BCRP, and OATP1B1 activity levels, which transfer drugs into or out of the cells. Previous studies have demonstrated that P-gp, BCRP, and OATP1B1 play a crucial role in the absorption, distribution, and excretion of LOS *in vivo* (Pei et al., 2018; Ripperger et al., 2018; Shin et al., 2020). However, certain other drug transporters may participate in the metabolism of LOS in rat. Therefore, SMI can reduce the elimination of LOS, which is partially attributed to the inhibition of CYP3A4, CYP2C9, and of the drug transporters (P-gp, BCRP, and OATP1B1). While, the concentration of EXP3174 did not change irrespective of the SMI dose, which could be attributed to the combined effect of the reduced conversion of LOS to EXP3174 and to the reduced efflux of EXP3174 in the hepatocytes. In addition, the inhibitory effects of SMI on other CYP enzymes (except for CYP3A4 and CYP2C9) can provide evidence and a new direction for follow-up studies based on the interaction between this herbal medicine and other drugs.

An increased number of studies have selected monomer mixtures or original components of herbal medicines to explore HDI. Recent *in vitro* and *in vivo* studies on HDI were mostly performed using drug monomers. However, a monomer may be the substrate of multiple CYPs and drug transporters. In addition, the ultimate effects on CYPs enzymes and drug transporters, including HDI, were the result of the combined effects of multiple monomers that originate from herbs. Therefore, in order to simulate the situation occurring in the human body, monomer mixtures or original herbal components were selected for exploring HDI. Our previous *in vitro* study demonstrated that Shexiang Baoxin Pill could inhibit various CYPs and the enzyme OATP1B1 at varying extents (Shen et al., 2016). Similarly, Pao et al. evaluated the effects of 50 types of Chinese herbal medicines on CYP3A4 *in vitro* and *in vivo* so as to predict the possible HDI (Pao et al., 2012). Fung et al. found that the extracts of red yeast rice could interact with Verapamil by inhibiting the activity of CYP3A4 and increasing the activity of P-gp (Fung et al., 2012).

We observed for the first time that SMI decreased the protein expression of CYP3A4 and P-gp, but not of CYP2C9. Previous studies on the effects of SMI on CYP expression are inconclusive. However, numerous studies have shown that herbs or monomers of herbs can affect the protein or mRNA expression levels of CYP3A4, CYP2C9, and P-gp. He et al. demonstrated that ChaihuShugan powder, an anxiety treatment prescription, could increase the protein and mRNA expression of CYP3A4 by activating the progesterone X receptor (PXR) pathway in a mouse model of depression (He et al., 2019). SA-B and Tan IIA, both of which are components of Salvia Miltiorrhiza, have demonstrated opposite effects on the protein expression levels of CYP3A4 and CYP2C9 *in vivo* (Wang et al., 2016).

SMI could inhibit the protein expression of CYP3A4 and P-gp for the following reasons. Firstly, the single nucleotide polymorphism (SNP) of CYP3A4 and P-gp was a considerable factor for the difference noted in protein expression. However, the presence of SNPs cannot explain all the protein expression differences noted between various individuals and accounts for only 10–20% (Rahmioglu et al., 2011). Secondly, SMI affects PXR and androsterane receptor (CAR), which are recognized sensors that can combine exogenous chemicals with different structures (Elmeliegy et al., 2020). The mRNA and protein expression levels of CYP3A4, CYP2C9, and P-gp are regulated by CAR and PXR. However, the results indicating that the protein expression of CYP2C9 did not decrease following SMI treatment were contradictory (Wang et al., 2012). Certain herbs or monomers of herbs can affect the expression levels of CYP3A4 and P-gp by regulating PXR or CAR. Yang et al. indicated that Ginseng extract could suppress the mRNA and protein expression levels of CYP3A4 induced by PXR in Sprague-Dawley rats (Yang et al., 2019). Kratom, a native herb, which grows in Southeast Asia, can increase the protein expression of CYP3A4 and P-gp by activating PXR in human hepatocellular carcinoma (HepG2) cells (Manda et al., 2017).

The effects of Ginsenoside Rg1, Ginsenoside Re, Ginsenoside Rb1, and Schisandrin A on CYP3A4, P-gp, and CYP2C9 were also reviewed (Table 4). The components originating from Ginseng indicated contradictory effects on CYP3A4, CYP2C9, and P-gp. Inhibition, absence of effect, and induction were noted with regard to the aforementioned enzymes. These effects may be attributed to the different times of administration, different types of Ginseng (Ginseng, red Ginseng, fermented red Ginseng), and different extraction methods of the herbal medicines used (Yang et al., 2019). Both Schisandrae chinensis extract and Schisandrin A indicated inhibitory effects on CYP3A4, CYP2C9, and P-gp. The constituents of Radix Ophiopogonis, which were rarely detected in SMI, were not discussed in the present study. Compared with the results of our study, we speculated that the inhibitory effect of SMI on CYP3A4, CYP2C9 and P-gp may originate from the combined effects of ginsenoside and Schisandrin A. In this study, we did not carry out LC-MS determination of various active components of SMI, therefore, we could not have a profound understanding of the interaction between SMI and LOS. In the next step, we will take monomers as the object to conduct a deeper exploration on the mechanism.

**TABLE 4:** Studies about effects of main components in SMI on CYP3A4, CYP2C9 and P-gp. HepG2, Human Hepatoellular Carcinomas; Caco-2, Human Colon Adenocarcinoma Cell Line; NA, no data.

Author	Time	Ingredient	Subject	Effects on CYP3A4	Effects on CYP2C9	Effects on P-Gp
Nu He He and Edeki, (2004)	2004	Ginsenoside Rb1	Human liver microsomes	Inhibition	Inhibition	NA
Miao Hao Hao et al. (2008)	2008	Ginsenoside Rb1、Rg1、Re	Human liver microsomes	Inhibition	Inhibition	NA
Jianbo Chen Chen et al. (2021)	2020	Ginseng saponins	Sprague-Dawley rats	Induction	Induction	NO
Christine Y. Malati Malati et al. (2012)	2012	Panax ginseng	Healthy volunteers	Induction	NA	NO
Sook Jin Seong Seong et al. (2018)	2018	red ginseng extracts	Healthy volunteers and human hepatocytes	NO	NO	NO
Min-Gul Kim Kim et al. (2016)	2016	fermented red ginseng	Healthy volunteers	NO	NO	Inhibition
Wei-Liang Li Li et al. (2012)	2011	Schisandrin A	Rat hepatic microsomes	Inhibition	NA	NA
Xiao Ling Qin Qin et al. (2013)	2013	schisandrol A	Sprague-Dawley rats	Inhibition	NA	Inhibition
Y.-F. Cao Cao et al. (2009)	2010	schizandrin	Human liver microsomes	Inhibition	NA	NA
C.-K. Wan Wan et al. (2010)	2010	schisandrol A	HepG2	Inhibition	NA	Inhibition

The current study contains certain limitations. Firstly, the safety evaluation following the combination of SMI and LOS was not conducted, which requires further determination. Secondly, the limitations of the clinical use of SMI were evident. Since SMI is administered intravenously, its combination with LOS is only possible during hospitalization. In any case, the current study provided proof for decision-making during hospitalization to a certain extent. Thirdly, Numerous studies have shown that there are some differences betwen rat and human in isoform composition, expression and catalytic activities of drug-metabolising enzymes, therefore, clinical trials examining the combination of SMI with LOS need to be conducted (Martignoni et al., 2006; Hussner et al., 2021).

In conclusion, SMI improved the antihypertensive efficacy of LOS in the L-NAME-induced hypertension rat model by increasing the concentration of LOS, while leaving the concentration of EXP3174 intact. SMI affected the pharmacokinetic properties of LOS by decreasing the elimination of LOS. These effects might partly be attributed to the inhibition of the activities of CYP3A4, CYP2C9, and of the drug transporters (P-gp, BCRP, and OATP1B1) by SMI, which need further scrutiny.

## Data Availability

The raw data supporting the conclusions of this article will be made available by the authors, without undue reservation.
