# Supplementary material for: Evaluation of Potential Herb-Drug Interactions Between Shengmai Injection and Losartan Potassium in Rat and In Vitro
Source: Front Pharmacol. 2022 Apr 20;13:878526. doi: 10.3389/fphar.2022.878526 (PMC9065348; doi:10.3389/fphar.2022.878526)
Supplement: Supplementary file 1 [file DataSheet1.PDF]

1 Supplementary data

1.1 LC-MS methodology and method validation in rat and *in vitro*

1.1.1 LC-MS methodology of LOS and EXP3174 in plasma of Wistar rat

1.1.1.1 Chromatography and mass spectrometry

The chromatographic analysis of LOS, EXP3174, and irbesartan was performed on a Dionex Ultimate 3000 High-pressure liquid chromatography (Dionex, USA) system. The analysis was performed at room temperature, and an Acquity UPLC®HSS T3 column (2.1 mm×100 mm, 1.8 μm, Waters, USA) was used. The mobile phase included 0.1% formic acid aqueous solution (C) and acetonitrile (D) at a flow rate of 0.3 ml/min. The proportion of phase B in the mobile phase was reduced from 40% to -5% at 0-2.0 min. Subsequently, it remained stable to 5% at 2.0-4.0 min and it was increased to 40% at 4.0-5.0 min. The column temperature was adjusted to 45℃ and the injection volume was 3 μl.

The mass spectrometry analysis of LOS, EXP3174and irbesartan was performed on Q/Exactive Quadrupole/Electrostatic Field Track Well High Resolution Mass Spectrometer (H-ESI II, Thermo Fisher Scientific, USA) in positive ion detection mode. The ion source was HESI. The sheath gas flow rate was 35 AU, whereas the auxiliary gas flow rate was 10 AU. The capillary temperature was 320℃. The S-lens RF level was 50 and the auxiliary gas temperature was 300℃. The spray voltage was +3.2 KV/-2.8 KV. The detection method was adjusted to full MS, the resolution was 70,000, and the scanning range was set at 300 ~ 500 (m/z).

1.1.1.2 Preparation of reference solution

Appropriate amounts of LOS, EXP3174, and irbesartan were dissolved in acetonitrile solution. The final concentrations of 1 mg/ml LOS and EXP3174 standard stock solution and 100 μg/ml irbesartan internal standard solution were prepared following dissolution by ultrasound. An appropriate volume of LOS and EXP3174 standard stock solution was diluted with methanol to a mixed standard solution of LOS and EXP3174 with a final concentration of 50, 25, 12.5, and 6.25 μg/ml. An appropriate amount of irbesartan internal standard stock solution was diluted with acetonitrile solution to prepare a final concentration of 1 μg/ml irbesartan acetonitrile standard solution.

1.1.1.3 Sample preparation

A total of 100 μl plasma sample was mixed with 300 μl acetonitrile standard solution of irbesartan thoroughly, following centrifugation at 12,000 rpm at 4°C for 10 min. A total of 150 μl supernatant was transferred to the sample bottle. The aforementioned chromatographic and mass spectrometry conditions were used for the determination of the samples.

1.1.2 LC-MS methodology for determining the activity levels of CYP enzymes and the transporter OATP1B1

1.1.2.1 Chromatography and mass spectrometry experimental conditions

The chromatographic analysis was performed on a liquid chromatography system (Shimadzu LC20, Japan). The chromatographic columns used were the DikmaInspire C18 column (50

mm×2.1 mm, 5 μm) and the Phenomenex SynergiTM Hydro-RP 80A column (30 mm×2 mm, 4 μm,). The mobile phase used included 0.1% formic acid aqueous solution (A) and 0.1% formic acid in acetonitrile solution (B). The proportion of phase B in the mobile phase was adjusted from 90% to 30% at 0-0.70 min, 30% to 5% at 0.70-0.71 min, 5% at 0.71-1.20 min, 5% to 90% at 1.20-1.21 min, 90% at 1.21-1.50 min, 90% to 30% at 1.50-1.70 min, 30% at 1.70-2.01 min, and 30% ~ 80% at 2.01 ~ 2.30 min. The column temperature was 45℃, the injection volume was 5 μl, and the volume flow rate was 0.45 ml/min.

The mass spectrometry analysis was performed on an AB Sciex API4000 mass spectrometer/liquid mass spectrometer (Applied Biosystems, INC., API4000). Electrospray ionization source (ESI) and multiple reaction monitoring mode were used. In the ESI^+ mode, the collision, curtain, atomizing and auxiliary gas pressures were 41.37, 137.90, 344.75, and 344.75 kPa, respectively. The ion voltage was 5,000 V and the ion injection temperature was 500℃. In the ESI− mode, the collision gas pressure was 55.16 kPa, the curtain gas pressure 137.90 kPa, the atomizing gas pressure 344.75 kPa and the auxiliary gas pressure 344.75 kPa. The ion voltage was −4,500 V, and the ion injection temperature was 500℃. The mass spectrometry conditions and ions of the target metabolites are shown in ***supplemental Table 2***.

1.1.2.2 Preparation of reference substance solution

All reference solutions were dissolved in methanol. Paracetamol, hydroxybupropion, 6β-hydroxytestosterone, 4-hydroxydiclofenac, 4-hydroxymephenytoin, and dextrorphan were prepared as 10 mmol/l reference substance stock solutions. N-deethyl amodiaquine and 17-β-glucoside-estradiol were prepared as a 1 mg/ml reference substance stock solution; buspirone, tolbutamide, and terfenadine were prepared as 10 mmol/l internal standard stock solutions; verapamil was prepared as a 1 mg/ml internal standard stock solution.

1.1.2.3 Sample preparation

A total of 50 μl was added to the standard curve, quality control, and test samples; a total of 50 μl blank matrix was added to the blank and blank quality control samples and 50 μl without the internal standard was added to the upper limit of quantification. Subsequently, 200 μl internal standard working solution (buspirone 10.0 nmol/l, verapamil 0.400 ng/ml, tolbutamide 20.0 nmol/l, and terfenadine 10.0 nmol/l) was added to the standard curve, quality control, blank quality control, and test samples respectively. A total of 200 μl methanol was added to the blank and the upper limit of quantification samples. All samples were vortexed for 10 min and centrifuged at 4°C, at 4,000 r/min for 10 min. A total of 200 μl supernatant was transferred to a 96-well polypropylene plate and the samples were injected for determination.

1.1.3 Method validation of LC-MS *in vivo* experiments

1.1.3.1 Specificity examination

A total of 3 μl reference, sample, and negative control solutions (blank serum) were obtained for LC-MS measurement. As shown in ***supplementary Figure 1***, the results indicated that the peak shape of LOS and EXP3174 was optimal, and the negative control (blank serum) did not interfere with the position of the reference substances (LOS and EXP3174).

1.1.3.2 Linear relationship and matrix effect

The two target metabolites were examined using the conditions of selected sample processing, chromatography, and mass spectrometry. No absolute matrix effect was noted at the three

concentration levels (low, medium, and high corresponding to 10, 40, and 100 ng/ml). The quantification and detection limits of the target metabolites were calculated with 10 times the signal-to-noise ratio. The concentration of each metabolite was denoted by the abscissa (X), whereas the ratio of the peak area of each metabolite to the internal standard by the ordinate (Y). A linear regression equation was established. As shown in ***Supplementary Table 3***, the linear correlation coefficients (R2) of the two target metabolites were both higher than 0.994, the quantification limit was 0.6 ng/ml, and the detection limit was 0.2 ng/ml.

1.1.3.3 Precision and stability

As shown in ***Supplementary Table 4***, the RSD of the low, medium, and high concentration (10, 40, and 100 ng/ml) quality control samples was calculated to determine the precision. The RSD of the lowest concentration quality control sample was within 20%, whereas the RSD of the medium and high-concentration quality control samples was within 15%, indicating optimal precision. Low, medium, and high concentrations of LOS and EXP3174 serum samples were placed at room temperature for 12 h. Following repeated freezing and thawing at -80°C for 1 week, the RSD was estimated to be less than 15%, indicating optimal stability of the samples.

1.1.3.4 Extraction recovery rate

The comparison of the ratio between the chromatographic peak area of the samples following the extraction and the chromatographic peak area of the sample without extraction led to the calculation of the extraction recovery rate during the sample pretreatment process. The RSD of the extraction recovery rate of the quality control samples corresponding to the low, medium, and high concentration samples (10, 40, 100 ng/ml) was within 15%, indicating that the extraction recovery rate during the sample pretreatment process was the same at different concentrations.

1.1.4 Method validation of the LC-MS *in vitro* experiment

1.1.4.1 Linearity range and matrix effect

Under the selected chromatographic and mass spectrometry conditions, the 8 target metabolites exhibited no absolute matrix effect at the low, medium, and high concentration levels; the quantification limits of the target metabolites were calculated with 10 times the signal-to-noise ratio and detection limit. The concentration of each metabolite was denoted by the abscissa (X) and the ratio of the peak area of each metabolite to the internal standard as the ordinate (Y). A linear regression equation was established. As shown in ***Supplementary Table 5***, the linear correlation coefficients (R2) of the 8 target metabolites were all higher than 0.994, the quantification limit was 10 mmol/l, and the detection limit was 10 mmol/l.

1.1.4.2 Precision test

The precision was investigated by calculating the RSD changes between quality control samples of the low, medium, and high concentrations (1.2, 40, 80 mmol/l). The RSD of the low concentration quality control sample was within 20%. The RSD of the quality control samples of the medium and high concentration samples were within 15%, indicating optimal precision. The results are shown in ***Supplementary Table 4***.

1.1.4.3 Extraction recovery rate

By comparing the ratio between the chromatographic peak area of the sample following the extraction and the chromatographic peak area of the sample without extraction, the extraction

recovery rate during the sample pretreatment process was investigated. The RSD of the extraction recovery rate of the low, medium, and high-concentration quality control samples was within 15% indicating that the extraction recovery rate during the sample pretreatment process was the same at different concentrations. The results are shown in ***Supplementary Table 4***.

1.2 Production method of SMI

After the extraction, concentration and purification of 100g red ginseng (*Panax ginseng* C.A.Mey. after processing), add aquae pro injectione (170512, 170515, 170517, 170519 and 170522; Shanghai Hutchison Pharmaceuticals, Shanghai, China) to 200ml, then made it into red ginseng precipitate solution through a series of processes.

312g *Ophiopogon japonicus* (Thunb.) Ker Gawl.was extracted, concentrated and purified by adding aquae pro injectione to 200ml, then made it into ophiopogon japonicus precipitate solution.

Collect 150ml Schisandra chinensis (Turcz.) Baill. distilled liquid by 156g *Schisandra chinensis*. The residue was extracted, concentrated and purified. After adding aquae pro injectione to 150ml, *Schisandra chinensis* precipitate solution was prepared by a series of processes.

The above red ginseng precipitate solution (200ml), ophiopogon japonicus precipitate solution (200ml), schisandrae chinensis distillate (150ml) and schisandrae chinensis precipitate solution (200ml) were combined. After the purification process, then add aquae pro injectione to 1000 ml.

2 Supplementary Figures and Tables

2.1 Supplementary Figure

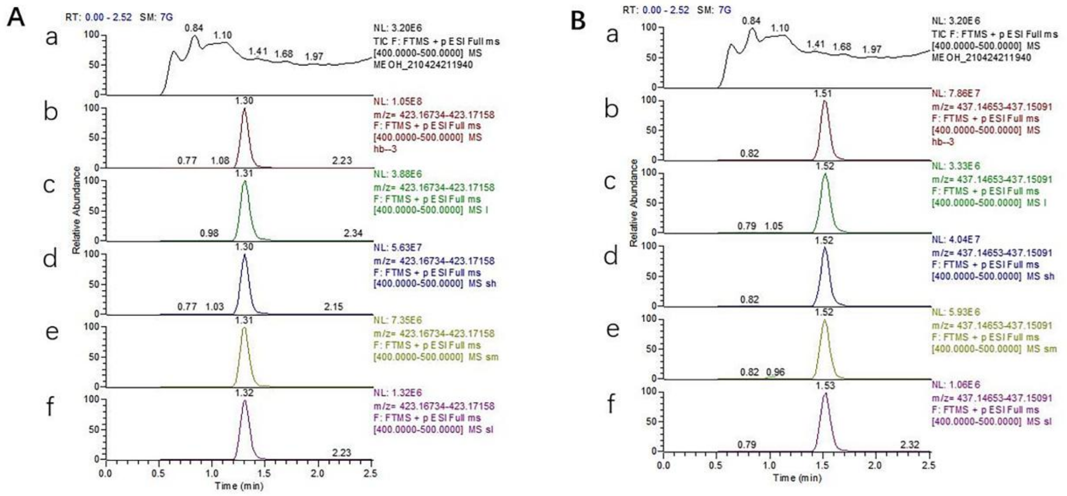

Supplementary Figure 1. Ion flow chromatography of losartan potassium(**A**) and EXP3174(**B**); **a** indicates blank serum; **b** indicates reference solution; **c** indicates samples of LOS group; **d** indicates samples of SMI-H+LOS group; **e** indicates samples of SMI-M+LOS group; **f** indicates samples of SMI-L+LOS group.

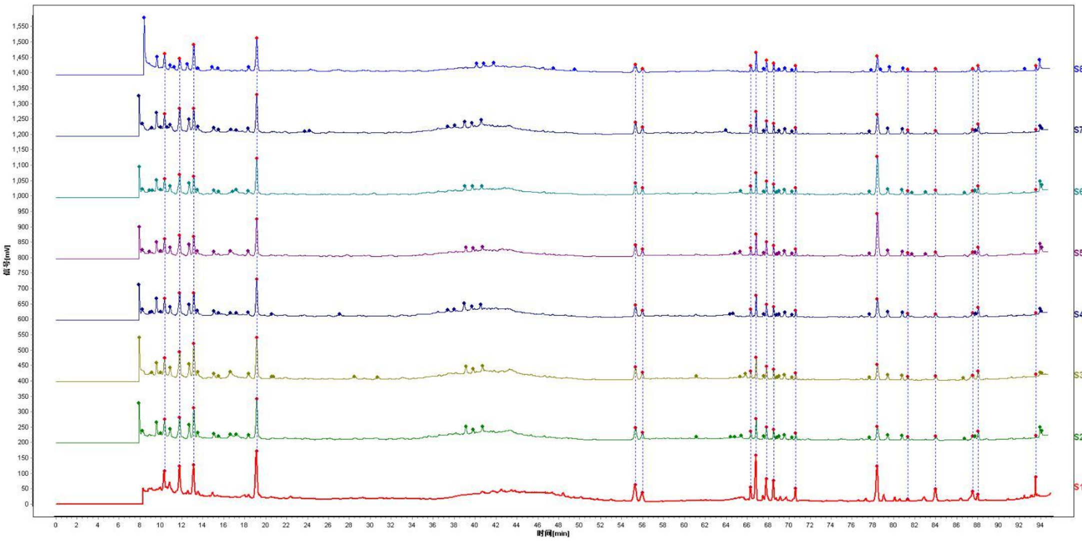

Supplementary Figure 2. Fingerprint of Shengmai Injection. S1: National standard (China); S2-S7: H18050201, H18050202, H18050301, H18050302, H18050401, H18050402; S8: H17050402 (Production batch number of SMI in this experiment).

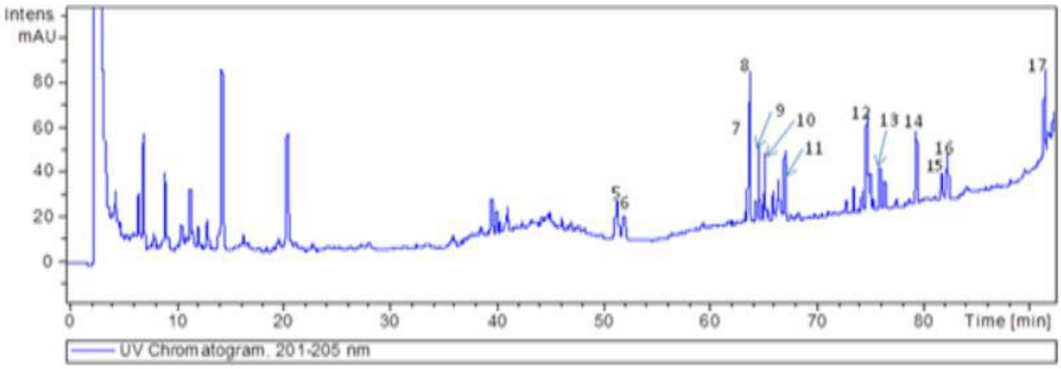

Supplementary Figure 3. Characteristic peaks of SMI assay by HPLC. 5: ginsenoside Rg1, 6: ginsenoside Re, 7: ginsenoside Rf, 8: ginsenoside Rb1, 9: ginsenoside Rc, 10: ginsenoside Rh1, 11: ginsenoside Rd, 12: schisandrin, 14: unknown (278 kD), 15 (16): ginsenoside Rg5 (ginsenoside Rk1), 17: ginsenoside Rh3.

2.2 Supplementary Table

Supplementary Table 1 Inhibitors, substrates and metabolites of CYP450 subenzymes.

CYP450 enzyme	substrate (final concentration, µM)	inhibitor (final concentration, µM)	metabolite
**CYP1A2**	Phenacetin (30)	α-Naphthoflavone (30)	Acetaminophen
**CYP2B6**	Bupropion (100)	Thiotepa (200)	Hydroxybupropion
**CYP2C8**	Amodiaquine (1.5)	Quercetin (100)	N-desethyl amodiaquine
**CYP2C9**	Diclofenac Sodium (25)	Sulfapyrazole (10)	4-hydroxydiclofenac
**CYP2C19**	S-Mephenytoin (50)	Ticlopidine (5)	4-hydroxymephenytoin
**CYP2D6**	Dextromethorphan (8)	Quinidine (10)	Dextrorphan
**CYP3A4**	Testosterone (100)	Ketoconazole (5)	6β-hydroxytestosterone

Supplementary Table 2. Target metabolic product spectrum conditions and ions.

Target metabolites	tR/min	Precursor ion	Production (m/z)	De-clustering	Collision
Acetaminophen	1.50	152.10	110.10	45	22
Hydroxybupropion	1.50	256.20	238.20	45	35
6β-hydroxytestosterone	2.30	305.40	269.30	70	20
4-hydroxydiclofenac	1.50	312.20	230.20	45	48
4-hydroxymephenytoin	1.50	235.20	150.10	70	25
Dextrorphan	1.50	258.30	157.20	113	52
N-desethyl amodiaquine	1.50	328.40	283.10	80	25
17β-glucoside-estradiol	1.50	447.50	74.90	−100	−10

Supplementary Table 3. Linear equation, correlation coefficient, detection limit, quantification limit and matrix effect of losartan potassium and EXP3174.

Compound	Linear equation	R^2	Linear range/ (ng.mL^{-1})	detection limit/ (ng.mL^{-1})	quantitation limit/ (ng.mL^{-1})	Matrix effect (%)
LOS	Y=0.0057 X+0.0438	0.998	0.6～625	0.2	0.6	95.42±7.93
EXP3174	Y=0.0048 X+0.0325	0.999	0.6～625	0.2	0.6	96.55±8.23

Supplementary Table 4. Extraction recovery rate and precision of 10 target metabolites *in vivo* and vitro.

Compound	Low-concentration quality control sample		Medium-concentration quality control sample		High-concentration quality control sample		Daytime precision
	Recovery rate	RSD	Recovery rate	RSD	Recovery rate	RSD	
Acetaminophen	103.20	7.20	101.56	7.02	106.21	15.03	71.62
Hydroxybupropion	93.27	2.46	93.72	6.09	92.79	11.72	11.89
6β-hydroxytestosterone	96.28	1.72	104.76	3.85	113.12	10.58	4.93
4-hydroxydiclofenac	105.63	8.83	103.18	4.61	102.87	12.19	8.71
4-hydroxymephenytoin	102.98	5.10	105.82	4.56	90.36	10.83	12.47
Dextrorphan	99.34	7.21	94.36	4.32	93.24	7.26	7.52
N-desethyl amodiaquine	82.76	8.99	74.21	4.75	68.85	5.01	8.59
17β-glucoside-estradiol	82.82	8.98	74.18	4.80	68.92	4.90	8.93
LOS	92.0	3.4	89.3	7.1	96.4	4.2	4.07
EXP3174	105	3.0	88.6	6.0	108.0	6.5	3.83

Supplementary Table5. Linear equation, correlation coefficient, limit of detection, limit of quantification and matrix efficiency of 8 target metabolites *in vitro*.

**Compound**	**Linear equation**	R^2	**Linear range/** （ng.mL^{-1}）	**detection limit/** （ng.mL^{-1}）	**quantitation limit/** （ng.mL^{-1}）	**Matrix effect** (%)
Acetaminophen	Y＝0.001 550 X＋0.009 53	0.998	100～25 000	100	100	93.40±6.28
Hydroxybupropion	Y＝0.000 498 X＋0.001 61	0.998	30～7500	7500	30	91.00±3.00
6β-hydroxytestosterone	Y＝0.000 293 X＋0.037 30	0.994	400～10 000	400	400	99.90±3.40
4-hydroxydiclofenac	Y＝0.000 924 X＋0.042 50	0.997	400～10 000	400	400	98.30±3.37
4-hydroxymephenytoin	Y＝0.003 990 X＋0.001 74	0.996	10～2500	400	10	91.30±4.68
Dextrorphan	Y＝0.002 690 X＋0.008 42	0.996	20～5000	20	20	96.90±4.41
N-desethyl amodiaquine	Y＝0.006 180 X＋0.009 91	0.999	40～10 000	40	40	99.10±3.39
17β-glucoside-estradiol	Y＝0.001 850 X－0.002 58	0.996	20～5000	20	20	95.80±3.33

Supplementary Table 6. Effects of SMI on CYP450 Enzyme Activity and IC50 (X±S, n=3)

**Groups**	**Dose**	**Relative activity (%)**						
**CYP450**	**(%)**	**CYP1A2**	**CYP2B6**	**CYP2C8**	**CYP2C9**	**CYP2C19**	**CYP2D6**	**CYP3A4**
NC	-	100.00±1.04	100.00±9.15	100.00±17.81	100.00±0.98	100.00±24.88	100.00±11.39	100.00±13.14
AC	-	14.41±0.69	15.22±0.46	34.34±1.84	15.59±0.52	30.13±5.23	9.13±0.29	3.86±1.96
SMI	0.1	101.65±4.29	110.18±3.51	97.48±7.49	98.79±1.34	113.26±19.49	102.42±2.54	112.13±31.07
	0.5	100.69±6.86	87.40±4.48	126.73±14.46	100.00±1.96	119.26±1.14	104.83±2.74	96.13±NA
	3.0	74.86±4.98	46.27±0.91	163.36±11.99	83.00±2.75	94.20±1.14	78.84±10.30	61.27±NA
	10.0	29.34±2.94	15.27±0.52	163.99±4.25	57.91±1.32	52.46±9.72	57.39±2.72	21.44±1.44
	30.0	15.00±1.33	14.69±0.81	46.23±1.23	32.68±1.46	21.01±4.99	25.65±0.63	2.93±0.42
IC50/%	-	6.12	2.72	10.00-30.00	14.31	12.96	12.26	3.72
95% CI/%	-	5.25-7.13	2.09-3.60	NA	13.1-15.4	8.06-16.8	9.89-14.8	2.38-7.24

Supplementary Table 7. Effect of SMI on the Relative Activities of MDR1, BCRP and OATP1B1 transporters and IC50(X ± S, n=3)

**Groups**	**Dose (%)**	**Relative activity (%)**		
**Drug**		**MDR1**	**BCRP**	**OATP1B1**
NC	-	100.00±3.82	100.00±7.53	99.90±2.04
AC	-	1.48±0.27	2.90±2.51	0.90±1.54
SMI	0.1	66.48±6.68	95.04±3.51	99.51±0.84
	0.5	17.22±2.32	60.93±3.51	99.27±2.08
	3.0	0.08±0.14	26.20±1.30	61.51±0.77
	10.0	0.99±1.17	7.15±0.30	2.64±0.16
	30.0	0	0.60±0.36	2.10±0.30
IC50/%	-	0.75	0.15	2.03
95% CI/%	-	0.118-0.191	0.630-0.887	1.18-3.49

Supplementary Table 8. Raw material information of SMI (H17050402)

Raw material	Lot number	Supplier	Content
red ginseng (*Panax ginseng* C.A.Mey. after processing)	H20140901<br>H20150903<br>H20150903-1	Jiju Shenye Co., Ltd., China	100g
Ophiopogon japonicus (Thunb.) Ker Gawl.	H2016090802	SPH Huayu Chinese Herbs, China	312g
Schisandra chinensis (Turcz.) Baill.	H2016101705-1<br>H2016111602	SPH Huayu Chinese Herbs, China	156g
		Made into 1000ml	

Supplementary Table 9. Concentrations of major components in SMI

Number of peak	Structure	Component	R1	R2	Concentration (ug/ml)
5	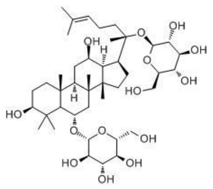	ginsenoside Rg1	Glc	Glc	295
6	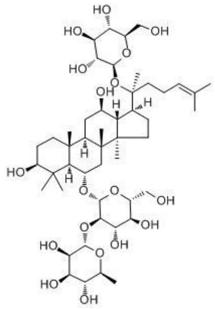	ginsenoside Re	Glc (2→1) Rha	Glc	182
8	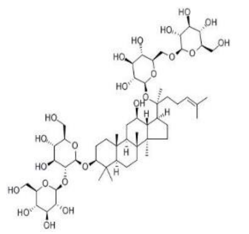	ginsenoside Rb1	Glc (2→1) Glc	Glc (6→1) Glc	366
12	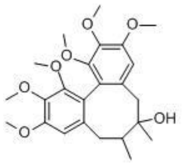	schisandrin	schisandrin		19.13
